# Buffalo Medical College

**Published:** 1848-11

**Authors:** 


*Buffalo Medical College.*—The preliminary dissecting term in this institution commences on the 1st inst. The lecture term commences on the last Wednesday of this month (Nov.)

The walls of the new edifice are erected, and the roof in progress of completion. In a previous notice of this structure, we inadvertently committed an error as respects its dimensions. Instead of being 160 ft. by 50 ft. as stated, it is 100 by about 60 feet.

